# Microbial Interaction as a Determinant of the Quality of Supply Drinking Water: A Conceptual Analysis

**DOI:** 10.3389/fpubh.2018.00184

**Published:** 2018-06-26

**Authors:** Syeda T. Towhid

**Affiliations:** Department of Microbiology, Jagannath University, Dhaka, Bangladesh

**Keywords:** municipal water distribution, microbial biofilm, waterborne diseases, deterioration of water quality, biofouling

## Abstract

This conceptual analysis elucidates the microbial interaction inside municipal distribution pipes, subsequent deterioration in the quality of the supply water, and its impacts on public health. Literature review involved a total of 21 original reports on microbiological events inside the water distribution system were studied, summarizing the current knowledge about the build-up of microbes in treated municipal water at various points of the distribution system. Next, original reports from the microbiological analysis of supply water from Bangladesh were collected to enlist the types of bacteria found growing actively. A schematic diagram of microbial interaction among the genera was constructed with respect to the physical, chemical, and microbiological quality of the supply water. Finally latest guidelines and expert opinions from public health authorities around the world are reviewed to keep up with using cutting-edge molecular technology to ensure safe and good quality drinking water for municipal supply.

## Introduction

Industrial revolution, urbanization, rapid increase in urban population, and increasing demand for expansion of public health infrastructures, increase in the demand for supply of drinking water from the municipality has led to complete change of the quality of lives in urban and sub-urban areas (1). Sustainable supply of safe drinking water is also a target to be achieved in the sustainable development goals 2030 (2). Therefore, maintaining the quality of the supply water is just as important as developing the infra-structures of water supply and conserving the sources of natural waters. The municipal water supply system acquires the surface water or aquifer water, treats the surface water physically (UV radiation or filtration) or chemically (chlorination), if necessary, and then distributes them through a network of pipes and overhead tanks to the points-of-use (homes, industries, public places, health care facilities, etc.). The quality and safety of the water at the receiving ends depend on the quality of the source from which it is acquired, the nature of treatment given in the municipal water treatment plant, the amount of residual disinfectant remaining in the water and the environments in the distribution network (pipes and overhead/underground reservoirs) (3). A good number of intrinsic and extrinsic factors determine the microbiological safety of the municipal (mains) water. Intrinsic factors include the length and duration of the treatment given to the source water, material, and length of the distribution pipes, total carbon, iron, lead, phosphate, and sulfate contents of the water, physical parameters of the water (pH, alkalinity, turbidity, hardness, conductivity), biological oxygen demand (BOD), chemical oxygen demand (COD), dissolved oxygen (DO), loose deposit accumulation inside the pipes, and the overhead tanks and microbes remaining in the supply water (4). All these factors might contribute to the quality deterioration potential (QDP) of the supply water as well (5). External factors also contribute to QDP. The deterioration of the quality of potable waters from distribution network emerged a decade ago when foul-odor or reddish color or incidences of waterborne diseases became a pressing issue (6). Subsequent investigation revealed that organisms from the natural water source, that survive the disinfection process, thrive inside the water distribution system, and interact among themselves as well as with the surface of the distribution pipes to form complex biofilms. This biofilm is able to deteriorate the safety and quality of the supplied water in more than one ways (7). Developed countries with full-scale mains distribution system employ task forces to study and control the in-process change of supply water throughout its passage in the distribution network.

Bangladesh, a nation of 170 million people, has 204 municipalities that supply treated or untreated surface water and groundwater in urban and sub-urban areas [Dhaka Water and Sewage Authority (DWASA) Annual Report 2015–16]^1^ The capital Dhaka contains the oldest and the largest pipe network for water supply (DWASA Annual Report 2012–13)^2^ Seasonal epidemics of waterborne diseases are common in Dhaka city (8). Few reports are found referring to the waterborne outbreaks at the beginning of monsoon and the foul quality of water, but the mechanism of water quality deterioration inside the supply network in Bangladesh is yet to find. This conceptual analysis summarizes information from the developed countries and predicts the possible events inside a Bangladesh supply network that poses health hazards to the consumers. The high incidences of morbidity and mortality from waterborne diseases call for re-evaluation of the surveillance, monitoring, and in-process control of the municipal water supply system. According to the annual report of Dhaka Water Supply and Sewage Authority (DWASA 2015–16), the organization supplied 2,450 million liters of water daily and in 2016 from four water treatment plants. There are 3,500 km of water lines connected to 361,938 household supplied from 38 overhead tanks, which also are points of biofouling. In addition, 1,643 hydrants moisturize the streets and highways. The Microbiology and Chemical Division of DWASA measures 50 parameters of the supply water to ensure safety and quality, but the emerging risks in mains water is not assayed (DWASA Annual Report 2012–13). DWASA follows the previous guidelines from WHO (4), which does not include the emerging biological and chemical risk factors known today. Despite the best efforts, risks are mounting from drinking water which common people consider safe to drink. This conceptual analysis brings to light the probable risk factors present in the water supplied by DWASA, so that modern techniques are introduced for water safety. The WHO puts emphasis on chemical residues and microbial interaction in drinking water (9) Risks associated with biofilm formation are enrichment of pathogens in the water, production of toxins, deterioration of the pipe material, release of antibiotic resistance genes, and supporting high-risk parasites such as *Cryptosporidium* and *Naegleria fowlerii* feeding off the biofilm (10).

This conceptual analysis attempts to predict the microbial interactions inside the water distribution pipelines, especially development of a biofilm consortium inside the pipes in Bangladesh so that an emerging risk to public health can be dealt with.

## Theoretical framework for the conceptual analysis

**Figure 1:**
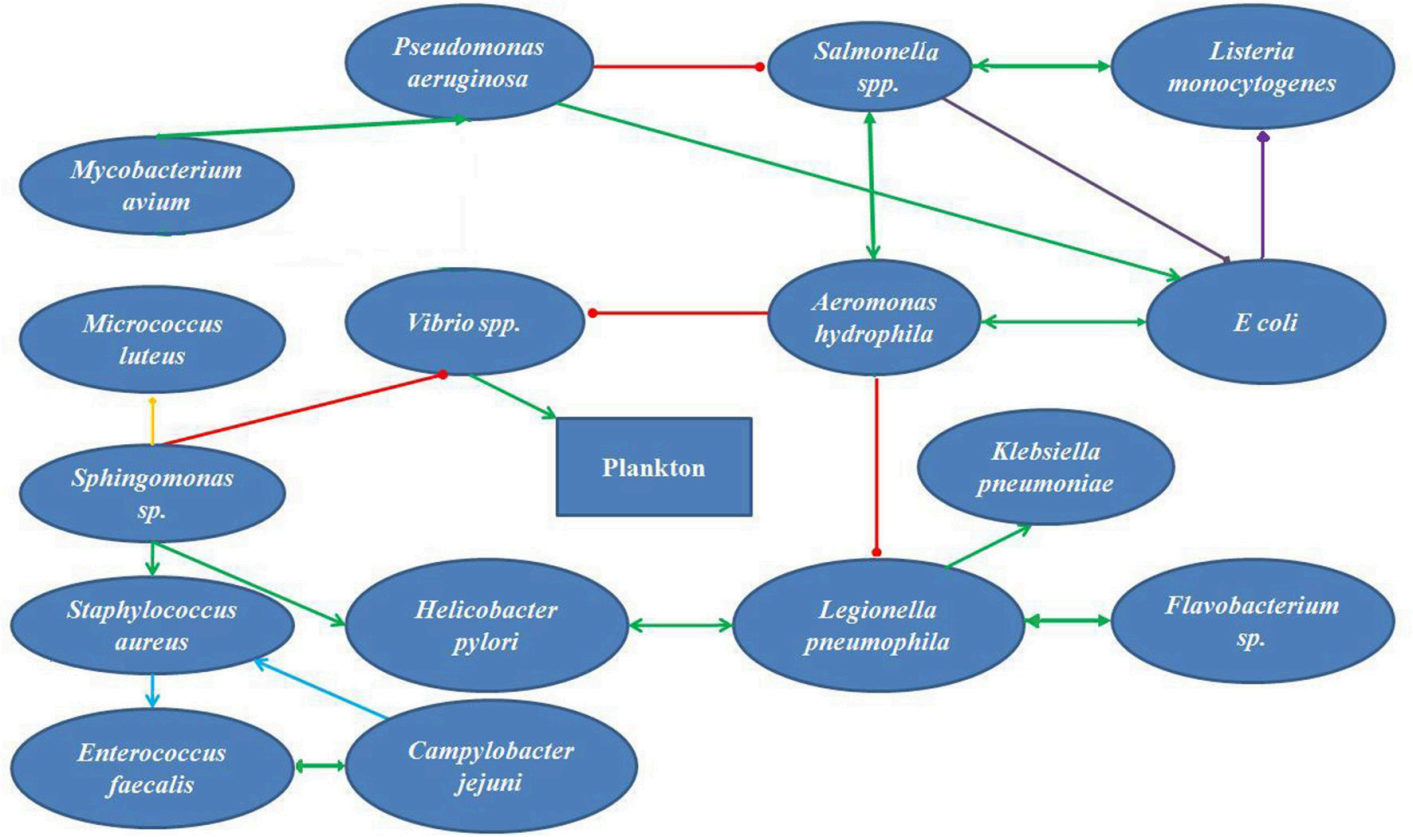
A schematic diagram for interaction among the bacteria found in supplied water in Bangladesh (cooperation 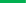, inhibition 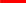, competition 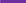, co-aggregation 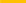, benefit to one species 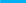).

Every natural environment has its own microbial community, that plays characteristic function depending on the interaction between each species in the community. The bacteria present in tap water are likely to represent the species present in the biofilms inside the distribution system. The original reports on bacterial isolates discovered from tap water around the world (Table 1) and in Bangladesh were enlisted (Table 2). The interaction between each pair of species in biofilm were studied from original articles on dual-species biofilm formation experiments. A theoretical diagram was constructed to hypothesize the probable interaction of the reported bacterial species in the biofilm consortium (Figure 1). Interactions between the bacteria and other common members of the biofilm in water distribution system in other countries were studied (Table 1) and most common organisms associated with any given member of the hypothetical biofilm was included in Figure 1 because protozoa, viruses, and worms constitute matured biofilms inside the water supply pipes and pose considerable threat to consumers. The impact of the hypothetical biofilm on corrosion of the water distribution pipes and deterioration of water quality was studied from reports on water quality maintenance from around the world.

**Table 1:** Original reports on biofilms inside municipal water distribution pipes.

**ID**	**First author**	**Year (references)**	**Country**	**Treatment of source water**	**Composition of Biofilm inside supply system**
1	Gottlich	2002 (7)	Germany	Filtration, coagulation sedimentation, chlorination	Bacteria, Fungi, worms
2	Penna	2002 (11)	Brazil	–	Bacteria, Fungi
3	Langmark	2005 (12)	Sweden, Australia	Chlorination UV treatment	Bacteria
4	Hageskal	2006 (13)	Norway	–	Fungi
5	Meier	2008 (14)	UK	Chlorination	Bacteria
6	Sammon	2010 (15)	Australia	Chlorination Residual chlorine 0.643 mg/L	Fungi, Bacteria, Archaea
7	Siquira	2010 (16)	Brazil	–	Fungi, mycotoxins
8	Li	2010 (17)	China	Conductivity 393 μS/cm, Sulfate 230 mg/L Chlorine 44 mg/L	*Galleonella, Sideroxydans, Acidothiobacillus, Thermomonas*
9	White	2012 (18)	USA	–	*Nitrospira, Sphingomonas, Hyphomicrobium*
10	Lee	2013 (19)	Korea	UV, Chlorination, DO 10 mg/L, volatile acetate	*Stenotrophomonas, Sphingomonas, Acidovorax, Microbacterium*
11	Shaw	2015 (20)	Australia	–	Bacteria, Cyanobacteria, Rhizobiales
12	Falkinham	2015 (21)	USA	–	Bacteria*, Acanthamoeba, Fungi*
13	Rozej	2015 (22)	Poland	–	*Proteobacteria, Bacteroidetes, Methylophilaceae, Geothrix*
14	Karkey	2015 (23)	Nepal	Chlorination 10–169 mg/L	Enterobacteriaceae, Clostridiales, Bacteroidetes
15	Abberton	2016 (24)	Ireland	–	*E. coli*
16	Ginige	2016 (25)	Australia	High ATP, minerals	*Bacteria*
17	Douterelo	2014 (26)	UK	Nitrate, Nitrite, TOC, phosphate, sulfate	*Flavobacterium, Bacteroidia*, Proteobacteria, Mollicutes, Spirochetes, Clostridia, Cyanobacteria
18	Prest	2016 (27)	Netherlands	–	Bacteria
19	Kooij	2016 (28)	Netherlands	10^7^ CFU/cc	*Legionella pneumophila, Vermamoeba*
20	Liu	2017 (5)	Netherlands	No treatment	Bacteria
21	Li	2017 (29)	China	Carbon filter, ozonation, Chlorination	Proteobacteria, *Bacteroidetes, Chlorfexi, Chlamydia, Actinobacter*, Cyanobacteria, Spirochaetes, Euryarchaeota

**Table 2:** Water parameters from Municipal Supply Water in Bangladesh.

**Parameter**	**WHO reference**	**DPHE reference**	**Values from independent studies from Bangladesh**
Source	–	–	Rivers
Temperature	–	–	29.8–31.7 (30)
pH	6.5–8.5	7	7.28–8.36 (30) 8.16 (31)
Conductivity	–	500 μS/cm	1,158 μS/cm (31)
Salinity	–	200 mg/L	–
Total dissolved solid	–	1,000 NTU	–
Turbidity	–	5 NTU	1.1 NTU (31)
Chloride ion	–	150–600 mg/L	–
Residual chloride	–	0.02 mg/L	–
Hardness	–	75 mh/L	–
Alkalinity	–	–	–
Nitrogen content	–	1 mg/L	–
Nitrite	0.3 mg/L	< 1 mg/L	–
Nitrate	50 μg/L	10 mg/L	0.51–3.66 mg/L (31) 8.5 mg/L (32)
Total aerobic bacterial count	–	–	*Salmonella, Shigella, Vibrio cholera, Aeromonas*, Fungi (33)
Total coliform	–	–	1–3 CFU/100 ml (33) *E. coli, Klebsiella* (30) TNTC (31)
Fecal coliform	–	–	*E. coli* TNTC (31)
BOD (5 day)	–	0.02 mg/L	0.55–1.74 mg/L (30)
COD	–	4 mg/L	–
Organic carbon content	–	–	–
Lead	0.01 mg/L	0.05 mg/L	0.05 mg/L (31)
Cadmium	0.003 mg/L	0.005 mg/L	–
Chromium	0.005 mg/L	0.05 mg/L	–
Arsenic	0.01 mg/L	0.05 mg/L	–
PCP	0	0.002 mg/L	–
Zinc	–	5 mg/L	–
Ammonia	–		–
Phosphate	0	6 mg/L	1.74–4.4 mg/L
Sulfate	0	400 mg/L	3.44–9.37 mg/L

## Literature review and inclusion-exclusion criteria

Literature review was done in three stages. First, Google Scholar, Pubmed, and the Cochrane Library were searched with keywords biofilm, water distribution system, municipality water pipes which returned 100 of results. Original reports that mentioned primary physico-chemical parameters of water and identified microbial species from biofilms developed inside municipal water supply pipes were included in this analytical report. Reports that did not mention the physico-chemical parameters or that did not identify biofilm members upto genus level were omitted. Gray literature was also omitted from literature review because information emerging from experiments done without strict adherence to established protocols might provide inaccurate information about biofilm composition. From the 21 relevant original reports on biofilms inside water distribution pipes listed in Table 1, the patterns of biofilms formed in temperate regions could be outlined. These studies carried out between 2002 and 2017 in Europe, the US, Australia, China, Korea, and Brazil show varied types of microbial populations in biofilms. Secondly, original reports on identification of microbial genera from municipal supply water in Bangladesh were searched in Google Scholar, Pubmed, and the Cochrane Library using search words Bangladesh, WASA supply water, tap water, microorganisms. A total of 11 original reports from Bangladesh satisfied the inclusion-exclusion criteria mentioned above and were included for constructing the schematic diagram of biofilm inside water supply pipes in Bangladesh (Table 2). Thirdly, Google Scholar, Pubmed, and the Cochrane Library were searched using keywords interaction of microbial species in biofilm to retrieve original reports of pair-wise microbial interaction in biofilms. A total of 13 articles that explored metabolic interaction between two bacterial species in an experimental biofilm in the laboratory control environment were included. Articles that could not conclude the specific interaction between dual- species were excluded from this study.

## Biofims inside water supply pipes

Microbial interactions differ depending on physico-chemical parameters of the natural waters. Temperature, pH, conductivity, turbidity, DO, type, and amounts of minerals, total organic Carbon (TOC), total Nitrogen, and biological/CODs are the non-biologic parameters that set the limits for microbial life (34). Different types of water treatment (desalination, decalcification, sedimentation) improve the physical quality of the source water (35). The distinct pattern of microbial interaction in aquatic environments is determined by the microbial population and the abiotic factors present. The microbes form and thrive in a biofilm through biomass transfer from the organic Carbon and microbial growth on any solid support (36). From the 21 relevant original reports on biofilms inside water distribution pipes listed in Table 1, the patterns of biofilms formed in temperate regions could be outlined. These studies carried out between 2002 and 2017 in Europe, the US, Australia, China, Korea, and Brazil show varied types of microbial populations in biofilms. The summer temperature in Europe and the North America is around 15 to 25°C and the natural population of the surface waters is diverse genera of bacteria, fungi, molds, bacteriophages, aquatic viruses, parasites, and in some cases Archaea. Brazil and Australia have an average summer temperature of 30°C, closer to the summer temperature of Bangladesh. Turbidity of the natural waters in the studies ranged from 0.4 to 58 NTU, closer to the range of the turbidity of surface waters around Dhaka city. The pH of the waters in the studies also falls within the range of Bangladesh river waters. The TOC content was stated to be 8.5 mg/L in one of the reports (31). The water distribution pipes composed of a wide range of materials depending on the soil type, depth, water pressure, flow, and retention pattern, intended life of the pipes etc. The pipe materials include unplansticized polyvinyl chloride (PVCu), chlorinated polyvinylchloride (PVCc), polyethylene-100 (PE-100), steel, polypropylene (PP), latex, polybutyrate (PB), copper, and high-density polyethylene (HDPE), all providing appropriate surface for biofilms to thrive (19). The type and duration of water treatment for disinfection reduces the numbers of organisms present in the natural waters. Most municipalities use multistep water treatments for reduction of unwanted minerals through filtration, flocculation, sedimentation, and disinfection (35). Chlorination and UV radiation are the most widespread methods for supply water disinfection. While UV radiation does not produce residual effect, chlorination is allowed to leave a threshold of 5 mg/L residual free chlorine so that any remaining pathogens are gradually killed on their way to the receiving end (37). However, resistant microbes can survive chlorination and establish complex biofilms inside the supply pipes, deteriorating the quality of supply waters (38). As evident from Table 1, the organisms that establish biofilms successfully inside a municipal distribution system range from pathogens (*Aeromonas hydrophila, Salmonella, Klebsiella, Pseudomonas aeruginosa, Legionella pneumophila, Escherichia coli*), opportunists (*Stenotrophomonas moltophilia, Mycobacterium avis* complex), toxin producers (Cyanobacteria) to non-pathogens that destroy the pipe material and cause biofouling (*Galleonella, Siderooxydans, Geothrix, Nitrospira*). The reports from temperate weather shows enrichment of molds in the biofilm (*Penicillium, Alternaria, Fusarium, Aspergillus, Mucor, Geotrichum, Botrytis*) (13), whereas reports from Australia and Brazil with higher temperatures show biofilms that are dominated by heterotrophic bacterial species (11, 16, 20, 25).

## Microbiological analysis of tap water from bangladesh

The best-studied water supply system in Bangladesh is in the capital Dhaka, which acquires water from rivers Meghna, Buriganga, Sitalakshya, and Turag, treats them in water treatment plans in Gandharbapur, Saidabad, Rupganj, and Pagla (39). They treat water with sedimentation and chlorination, test the water for safety and drinking quality and supply it through the pipes to overhead tanks, from which water goes into points of use. Mahbub et al. (40) reported finding live bacteria in more than 60% of the sampled tap water in their study, which exceeds the Bangladesh Standards (BDS 1240:2001) for the microbiological quality of water. The Bangladesh Department of Public Health and Engineering (DPHE) has set different set of standards for potable water in Bangladesh, which varies from the universal standards set by the World Health Organization (WHO) in many parameters. Coliforms and *E. coli* are frequently reported in supply water (31, 33, 41). Acharjee et al. (33, 42) had reported finding *E. coli, Klebsiella, Salmonella, Shigella, Vibrio, Aeromonas*, and fungi from supply water, indicating that these bacteria and molds survive disinfection procedures. When taken together with the quality of raw water with higher concentration of Iron and Arsenic, we find factors limiting certain kinds of microbes in the distribution system.

## Theoretical composition of biofilm inside the wasa water supply pipes in bangladesh

If we summarize the parameters reported from independent original studies, we can set the parameters of the supply water within the reported ranges. The temperature of the water in summer is around 30°C (30). The pH of the natural water varies between 7 and 8 and the DO ranges between 3 and 5 mg/L. The conductivity of the waters is 1,158 μS/cm, much higher than the reference value for natural waters (30). The turbidity of the water is 1.1 NTU. The nitrate concentrate was 8.5 mg/L, closer to the upper limit of nitrate concentration (33). The Iron concentration was 0.05 mg/L, and phosphate and sulfate concentrations were 4.4 and 9 mg/L, respectively. The tap water for domestic use contained 0.02 mg/L of residual Chlorine (43). Microbes that form biofilm within the water supply pipes must be organisms with their growth optimums within these ranges. According to Li et al. (29) the turbidity, ammonia concentration, nitrate content and TOC content of the water inside the supply system influence the nature and extend of biofilm formation. Pinto et al. (44) reported that the seasonal cycling of the biofilms inside the pipes correlated with seasonal temperature fluctuations. Bacteria that survive and develop biofilms under these conditions would be *E. coli, Shigella, Vibrio, Klebsiella, Salmonella, L. pneumophila, Flavobacterium, Sphingomonas, P. aeruginosa, Nitrospira, Actinobacterium, Acidobacterium, Aeromonas, Sphingobacterium, Mycobacterium avium, Bacteroidium, Clostridia, Spirochaetes, Acremonium, Cladosporium, Fusarium, Microbacterium, Stenotrophomonas. Penicillium* and *Aspergillus* are the most abundant molds in the natural waters in the tropics. In addition, Cyanobacteria could survive the parameters of Bangladeshi natural waters and contribute to the toxin production. The biofilm forming bacteria have different aspects regarding their roles on public health. *Legionella pneumophila, Salmonella, E. coli, Shigella, Vibrio, Klebsiella*, and *Clostridia* are pathogens that pose considerable threat to consumers. *L. pneumophila* causes life-threatening pneumonia and respiratory distress (28). *Salmonella, Shigella, Vibrio, Klebsiella*, and *E. coli* are waterborne agents of enteric diseases, often causing seasonal outbreaks in Dhaka city (8). Opportunistic waterborne pathogens include *M. avium* complex, *Stenotrophomonas maltophilia*, and *A. hydrophila* that can infect immune-compromised groups such as infants, adults, pregnant women, and people with underlying medical conditions (cancer, HIV/AIDS, etc.). *M. avium* causes pulmonary, soft tissue and lymph node infections (45, 46). *Stenotrophomonas maltophilia* causes respiratory infection in cystic fibrosis patients (47). *Aeromonas hydrophila* is an enteric pathogen infecting children and immunocompromised people (48). The aquatic biofilms have also been implicated in spread of drug-resistance genes. Talukdar et al. (41) had shown presence of extended spectrum beta-lactam (ESBL) *E. coli* and *qnrS* elements for quinolone resistance from tap water in Dhaka city. Biofilm microbes also produce metabolites and components that change the quality of drinking water. *Aspergillus* spores are allergenic (49). The odor in tap water often results from dimethyl polysulfides, produced by *Pseudomonas, Flavobacterium, Aeromonas*, and *Penicillium* (50). Sulfur oxidizers like *Acidobacterium* change the pH of the water and produce foul odor and taste (51). *Nitrospira* spp. are nitrite-oxidizing autotrophs, colonizing plastic surfaces (52). Metal oxidizing bacteria corrode metal pipes (53). All these information stress on the development of robust and sensitive analytical techniques for evaluation of water quality as well as revised maintenance procedures that would help reduce formation of biofilms inside municipal water distribution pipes. Figure 1 presents a simplified hypothetical diagram of the microbial interactions inside a municipal water supply pipe.

## Hypothetical interaction of microbial isolates from wasa water in bangladesh

Over the last decade, original report of microbiological analysis of tap water from Bangladesh mentioned isolating viable cells of coliforms and *E. coli* (40), *Klebsiella, Salmonella, Shigella, Vibrio, Aeromonas*, and fungi (33). Literature search on interaction of each pair of these microorganisms in a dual-species interaction helps to construct a hypothetical network of microbes in the water supplied by WASA (Figure 1). *Aeromonas hydrophila* showed positive interaction *Salmonella* spp. and *Listeria monocytogenes* (54, 55) *Vibrio* spp. (56), but is inhibited by *P. aeruginosa* at the planktonic phase of biofilm development (57). *Salmonella* competes with *E. coli* in biofilm (58). *E. coli* maintains cooperative interaction with *A. hydrophila* (59) and *P. aeruginosa* (60) in a biofilm. *P. aeruginosa* and *M. avium* mutually benefit each other (21). Vibrio spp., the major recruiter of plankton in biofilms (61) are supported by *A. hydrophila* (56) *Sphingomonas* sp. enhanced sustainance of *S. aureus* (62) and *Helicobacter pylori* in biofilm (63). *Sphingomonas* coaggregates with *Micrococus luteus* (64). Biofilm-forming potential *S. aureus* and *Enterococcus faecalis* are enhanced by the presence of *Campylobacter jejuni* (65). *Aeromonas hydrophila* inhibited *L. pneumophila* (66), but maintained mutually beneficial interactions with *H. pylori* (63), *Klebsiella pneumonia* and *Flavobacterium* (67, 68).

## Recommendations for risk alleviation

**Figure 2:**
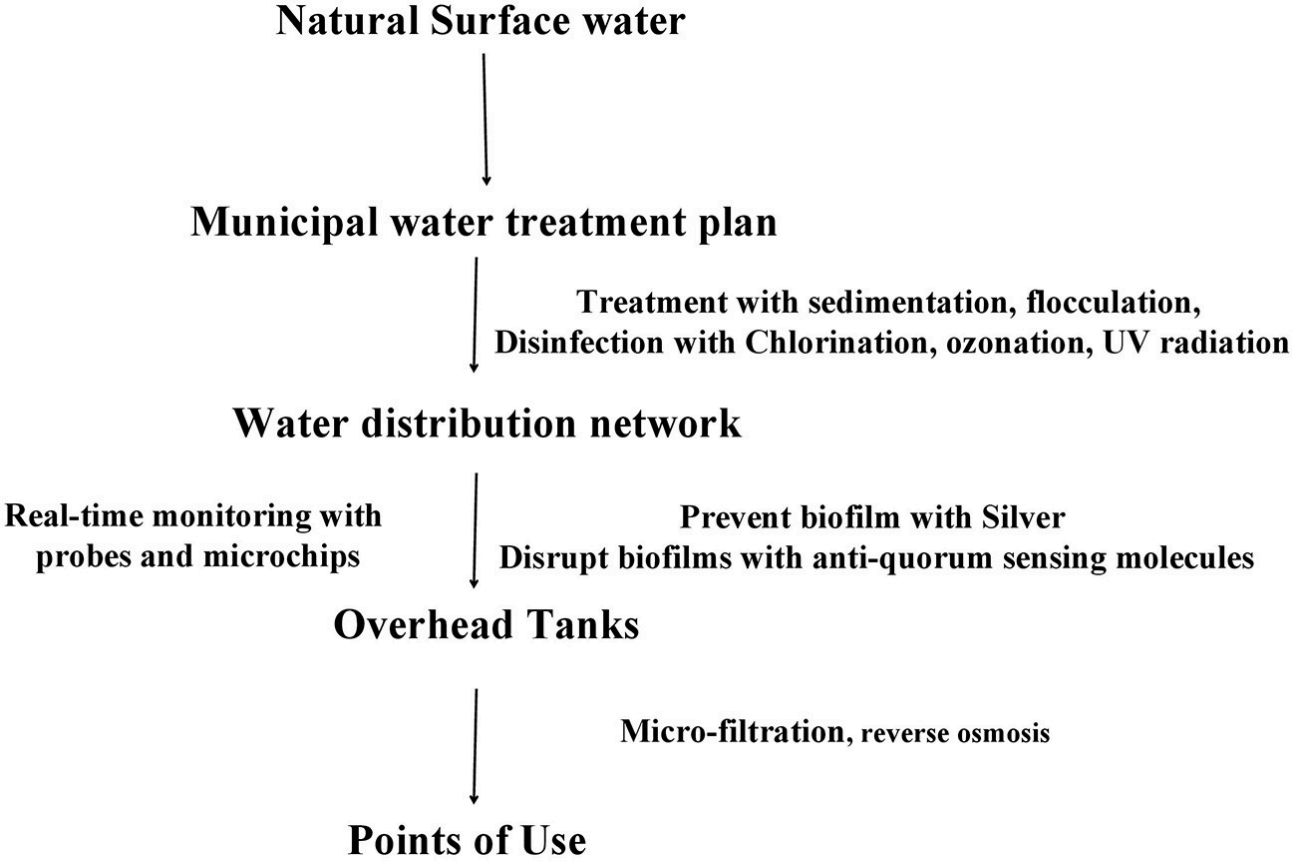
Diagram of preventive and disruptive measures for biofilm control and elimination at different stages of the municipal water supply network.

Public health microbiologists and engineers put much emphasis on preventing formation of complex biofilms inside the municipal water supply network. WHO guidelines suggests that national priorities should be determined while designing the public health infra-structure. A maximum of one water-borne infection per 10,000 consumers per year is an acceptable level for quality drinking water. The first consideration for building a good quality water treatment and supply network starts from identifying the source of water, identifying hazards quantitatively, constructing the pipe network, a cleansing regime should be in practice devised according to the material and the longevity of the pipes. Safe water framework from the WHO constitutes of an iterative method where quantitative microbial risk assessment method outlines the hazard analysis. If the hazards are identified quantitatively, control measures are sought for log removal of microbial hazard and for reducing chemical hazard (carcinogen, irritant, nitrification, and biocorrosion) to an acceptable level. Water pressure inside the pipes and the possibility of leakage along the pipes must be monitored closely. Alternate source of supply water for non-potable purpose could reduce the cost of municipal water treatment. Husna and Rahman (69) re-evaluated the necessity of rainwater harvesting in Dhaka city for industrial purpose. Real-time monitoring with commercial sensors and microchip-based devices should be in place to assess the physical, chemical, and microbiological quality of water. There are critical values of fluoride, nitrate, lead, chromium, arsenic, and pesticide concentrations in drinking water that counter biofilm formation inside the pipe distribution network, but these are detrimental to health at higher concentrations. Silvestry-Rodriguez et al. (70) reported that 100 μg/L of Silver can prevent biofilm formation in PVC and steel pipes but affects of elemental silver on consumer health needs to be studied. Hitzfeld et al. (71) discussed the effectivity of 1.5 mg/L of ozonation for 30 min with a residual concentration of 0.6 mg/L sufficiently maintaining Cyanobacteria under control, saving the water system from toxins. The United States Environment Protection Agency (72) put forward experimental techniques to disrupt chlorine-resistant biofilms by using anti-quorum sensing molecules, such as UW85 from *Bacillus cereus*. Another approach to getting safe drinking water is the treatment of municipal water at point-of-use, such as microfiltered water dispensation system or reverse osmosis water dispensers installed at hospitals, households, or nurseries (73). Figure 2 summarizes current and proposed preventing measures for biofilm build-up inside the water distribution pipes. National water safety plans should combine total quality management (TQM) and ISO 14001 and ISO9001 to ensure certified standards approved by independent group.

## Conclusion

The multi-faceted problem of development of biofilms inside the municipal water distribution pipe has not been addressed in Bangladesh yet. Microbial interactions get more complicated with virulence genes, especially antimicrobial resistance genes, that subjects consumers to even larger threat of antimicrobial resistance epidemic. The incidences of water-borne infections together with occasional deterioration in the quality of supply waters (odor, discoloration) calls for cutting-edge in-line real-time monitoring facilities with prompt interventions. Biological active Carbon (BAC) filters, granular active carbon (GAC) filter, UV lights, contact chlorine treatment, and an engineered storage and distribution might prove useful in improving water quality and safety.

## Author contributions

This manuscript enlists a single author, who reviewed scientific literature, formulated the study question and developed a theoretical model for the probable kind of microbial interaction inside water supply network in Bangladesh.

### Conflict of interest statement

The author declares that the research was conducted in the absence of any commercial or financial relationships that could be construed as a potential conflict of interest.

## Footnotes

^1^*Dhaka Water and Sewage Authority (DWASA) Annual Report 2015–16*. Available online at: http://dwasa.org.bd/annual-reports/2015-16.

^2^*Dhaka Water and Sewage Authority (DWASA) Annual Report 2012–13*. Available online at: http://dwasa.org.bd/annual-reports/2012-13.
